# Genetic basis of penicillin resistance of *S. aureus* isolated in bovine mastitis

**DOI:** 10.1186/1751-0147-54-69

**Published:** 2012-11-23

**Authors:** Arzu Funda Bagcigil, Suvi Taponen, Joanna Koort, Björn Bengtsson, Anna-Liisa Myllyniemi, Satu Pyörälä

**Affiliations:** 1 Department of Production Animal Medicine, Faculty of Veterinary Medicine, University of Helsinki, Helsinki, Finland; 2 Department of Microbiology, Faculty of Veterinary Medicine, University of Istanbul, Istanbul, Turkey; 3 Department of Basic Veterinary Sciences, Faculty of Veterinary Medicine, University of Helsinki, Helsinki, Finland; 4 National Veterinary Institute, SE-751 89, Uppsala, Sweden; 5 Finnish Food Safety Authority Evira, Helsinki, Finland

**Keywords:** Mastitis, *blaZ*, *Staphylococcus aureus*, Beta-lactamases, Penicillin-resistance Correspondence

## Abstract

**Background:**

The *blaZ* gene encoding penicillin resistance can be located either chromosomally or on plasmids. The aim of this study was to investigate the genetic relationships and to determine the location of the *blaZ* gene in *S. aureus* isolated in bovine mastitis in Finland and Sweden.

**Methods:**

Seventy-eight β-lactamase positive *S. aureus* isolates from bovine mastitis (34 from Finland and 44 from Sweden) were included in the study. The localization of *blaZ* gene was determined by Southern blotting. The *blaZ* genes of the isolates were sequenced and the sequences were translated to beta-lactamase proteins and further grouped as different protein signatures. The isolates and, as control, 33 Swedish and 36 Finnish beta-lactamase negative isolates were typed with pulsed-field gel electrophoresis (PFGE).

**Results:**

In 26 out of 34 Finnish isolates (76.5%) and in 25 out of 44 Swedish isolates (56.8%) the *blaZ* gene was localized on a plasmid. Six different protein signatures were found. One signature was found only in four Swedish isolates, but all other signatures were found both in Finnish and Swedish isolates. The PFGE results revealed a diversity of *S. aureus* clones. The protein signatures were not clearly associated with certain pulsotypes.

**Conclusions:**

The plasmid location of the *blaZ* gene was not statistically significantly more common in Finland than in Sweden, and hence does not explain the higher proportion of penicillin-resistant isolates of *S. aureus* causing bovine mastitis in Finland compared to Sweden.

## Background

Staphylococcal mastitis is a main problem of the dairy industry in many countries [1]. For mastitis with penicillin-susceptible *S. aureus*, benzylpenicillin is considered a first-line antibiotic due to its advantages compared with beta-lactamase stable penicillin [2]. Unfortunately, resistance to benzylpenicillin is common among mastitis-causing staphylococci, but varies largely between countries [3]. Low prevalences of penicillin-resistant *S. aureus* have been reported in Sweden (3.7 to 7.1%) and Norway (5.0 to 11.4%) [3-6] and in Canada (8.7%) [7]. High prevalences of penicillin-resistance have been reported for example in Estonia (61.4%). In England (36 to 46%) and in Korea (38.6 to 78.8%) [3,8,9]. In a prevalence survey in Finland, 52.1% of *S. aureus* isolates were penicillin-resistant [10]. In two other studies the level of resistance was lower, 22.9% and 25.0% [11,12]. It is, however, peculiar, why penicillin-resistance is clearly more common in Finland than in the neighbouring Nordic countries with similar conditions for milk production. One explanation could be the more common plasmid location of the *blaZ* gene and plasmid-mediated spread of penicillin-resistance.

Resistance to benzylpenicillin is mainly caused by the *blaZ* gene encoding production of beta-lactamases, which hydrolytically destroy beta-lactams [13]. The *blaZ* gene can be located chromosomally or on plasmids [14]. This type of penicillin resistance in *S. aureus* may thus emerge via two mechanisms: spread of resistant clones or through horizontal dissemination of mobile elements containing the *bla*Z gene [15,16]. Location of the resistance determinants on transferable elements generally promotes efficient spread [16]. In Denmark the *blaZ* gene of penicillin resistant *S. aureus* isolates has been predominately located chromosomally [17]. Diversity of the *blaZ* gene cluster can be studied by gene phylogenetic analysis [18,19] and be compared with published *blaZ* sequences, to find out possible relatedness.

The aim of this study was to investigate the location and diversity of the *blaZ* gene in *S. aureus* isolated in bovine mastitis in Finland and Sweden. In addition, the genetic relationships of *S. aureus* isolates were investigated. Our hypothesis was that location of the resistance gene on a plasmid would be associated with a higher prevalence of resistance. In addition, we investigated the diversity of *blaZ* sequences and compared the amino acid sequences of the beta-lactamase protein according to Olsen et al. [18].

## Methods

### Bacterial isolates

A total of 78 beta-lactamase positive *S. aureus* isolates from bovine mastitis (34 isolates from Finland and 44 isolates from Sweden) from previous surveys were included in the study. Out of the 34 isolates from Finland, 15 were collected in 2001 from subclinical mastitis [10] and 19 were collected in 2005 from clinical mastitis [12]. Out of the 44 Swedish isolates, 18, 12 and 14 isolates were from 2001, 2002/2003 and 2005, respectively. The isolates from 2001 and 2005 were from subclinical/chronic mastitis and were collected from diagnostic submissions to the Swedish National Veterinary Institute. The isolates from 2002/2003 were collected in a specific study on acute clinical mastitis [5].

Sixty-nine beta-lactamase negative isolates from both countries (36 isolates from Finland and 33 isolates from Sweden) were included in the pulsed-field gel electrophoresis (PFGE) analyses for comparison. Fourteen Finnish isolates were from 2001 and 22 from 2005. Out of the 33 Swedish isolates; 18 isolates were from 2001 and 15 from 2002/2003. The 36 Finnish and 33 Swedish beta-lactamase negative isolates originated from the same surveys described above [5,10,12].

The reference strain *S. aureus* ATCC 29213 was used for both *nuc* and *bla*Z PCR assays as a positive control and also for the probe preparation for Southern blotting. A beta-lactamase-producing *S. aureus* strain SV 41 was used in Southern blotting as the control of plasmid localisation of the *bla*Z gene [18].

### Confirmation of the isolates and PCR detection of the *blaZ* gene

Species confirmation was performed using PCR amplification of the thermonuclease (*nuc*) gene with the primers; P-1: 5^′^ GCG ATT GAT GGT GAT ACG GTT 3^′^; P-2: 5^′^ AGC CAA GCC TTG ACG AAC TAA AGC 3^′^ as described previously [20]. After the confirmation of presence of the *nuc* gene, all the isolates were further examined for the *bla*Z gene by using primers (blaZ 1: 5^′^ TTA AAG TCT TAC CGA AAG CAG 3^′^; blaZ 2: 5^′^ TAA GAG ATT TGC CTA TGC TT- 3^′^) designed by Olsen et al. [18]. The PCR-amplified products were separated by electrophoresis in a 1.5% agarose gel in lx TBE buffer (pH 8.3; 0.09 M Tris, 0.09 M boric acid, 2.0 mM EDTA) and stained with ethidium bromide (0.5ug/ml final concentration), visualized in UV-light and image analysed on AlphaImager HP Digital Imaging System, Model DE-500 (Alpha Innotech Corporation, San Leandro, CA, USA).

### Location of the *blaZ* gene in penicillin-resistant strains

The beta-lactamase positive isolates were included in Southern blotting analysis for the detection of the location of the *blaZ* gene. The bacteria were cultivated in 10 ml of Trypticase soy broth (TSB), supplemented with 10% yeast extract and benzylpenicillin at a final concentration of 8 μg/ml [17].

### Purification and digestion of chromosomal DNA and plasmid DNA

DNA was purified using the Easy-DNA™ Kit for genomic DNA isolation (Invitrogen Life Technologies, Carlsbad, CA, USA) according to the manual. For lysis of bacterial cells, 10 μl of lysostaphin (10 mg/mL) and 7, 5 μl of lysozyme (20 mg/mL) were added and the suspensions incubated at 37°C for 45 min. The E.Z.N.A.™ Plasmid Miniprep Kit (Omega Bio-Tek. Inc., GA, USA) was used for the purification of plasmid DNA. Purified chromosomal and plasmid DNA were digested with one unit of *Eco*RI (Roche Diagnostic, Mannheim, Germany) and *Eco*RV (Roche Diagnostic) for 2 h and run on a 0.8% Tris–borate EDTA agarose gel. A Digoxigenin (DIG)-labelled DNA Molecular Weight Marker II (Roche Diagnostic) served to determine the size of the digested chromosomal fragments [18].

### Southern blotting

A probe (377 bp) for the *bla*Z gene was prepared by PCR amplification on the *S. aureus* ATCC 29213 using the primers P1 and P2. Probe DNA was purified using Easy DNA Kit (Invitrogen, K1800-01) and labelled with digoxigenin-11-dUTP using DIG PCR Probe Synthesis Kit (Roche Diagnostic). DNA bands from chromosomal and plasmid digests were transferred from agarose gels onto Nylon membranes (Roche Diagnostic) by capillary transfer following depurination in 250 mM HCl, denaturation in 0.5 M NaOH, 1.5 M NaCl and neutralization in 0.5 M Tris–HCl, 1.5 M NaCl steps, as previously described [21]. Blots were prehybridised for 2 h at 42°C in prehybridisation solution (DIG Easy Hyb; Roche Applied Science). Denatured probe was added and hybridised to membranes for 12 h at 42°C in a hybridization oven. Membranes were then washed twice for 5 min at room temperature in 2 × Saline Sodium Citrate (SSC) buffer containing 0.1% Sodium Dodecyl Sulfate (SDS), and then twice for 15 min at 65°C in 0.5 × SSC–0.1% SDS. Chromogenic detection method was used for the visualization of the probes on the blot.

### PFGE analysis

Both beta-lactamase positive and negative isolates (totally 147 isolates) were examined by PFGE as previously described by Salmenlinna et al. [22]. Briefly, *S. aureus* colonies from overnight cultures were incorporated into low melting agarose (2%) plugs. The DNA purification protocol included lysostaphin and proteinase K treatment, followed by washing steps with phenylmethylsulfonyl fluoride and TE buffer (10 mM Tris and 1 mM EDTA, pH 8.0). Genomic DNA was digested using *Sma*I at room temperature, overnight. PFGE was performed by clamped homogeneous electric field (CHEF) electrophoresis with a CHEF-DR III System (Bio-Rad Laboratories, CA, USA) in a 1% (w/v) SeaKem agarose gel (FMC BioProducts, USA) at 6 V/cm by two phases: phase 1 for 10 h, with the initial and final switching times of 5 s to 15 s; and phase 2 for 13 h, with 15 s to 60 s, respectively. A Lambda Ladder PFG marker (New England BioLabs, USA) was used as the molecular weight standard. Fragments ranging from 50 to 800 kb were included in the analysis. Gels were analysed using BioNumerics v. 4.61 software (Applied Maths, Kortrijk, Belgium), and cluster analysis was performed by Unweighted Pair Group Method with Arithmetic Mean (UPGMA) based on the Dice similarity coefficient, with optimization and position tolerance set at 1.5%. Fingerprints with more than three band shifts were interpreted as being of a different pulsotype and thus the strains genetically unrelated.

### Sequencing of the *blaZ* gene

The *blaZ* genes of the beta-lactamase positive isolates were sequenced in the Institute of Biotechnology, Helsinki. The primers used for sequencing of plasmid located *blaZ* were 486: GTTGCGAACTCTTGAATAGG and 531: AATTCCTTCATTACACTCTTGG [18]. The primers used for sequencing of chromosomally located *blaZ* were 1 F: TACAACTGTAATATCGGAGGG and 1R: CATTACACTCTTGGCGGTTTC [19]. The gene sequences were translated to amino acid sequences using Transeq Nucleotide to Protein Conversion (European Bioinformatics Institute). The *blaZ* nucleotide sequences and the corresponding beta-lactamase amino acid sequences were compared with published sequences, retrieved from the GenBank, using BioNumerics24.61 software (Applied Maths). The amino acid sequences were grouped into protein signatures (three or less deviations in amino acid composition) according to Olsen et al. [18].

### Statistical methods

Frequencies of plasmid versus chromosomal location of *blaZ* genes in isolates from Sweden and Finland, and in isolates from clinical versus subclinical mastitis were compared using Pearson’s chi-square test.

## Results

### Confirmation of the isolates

All 147 isolates included in the study were *S. aureus*, as they showed amplification products at approximately 270 bp for *nuc* gene. All the 78 beta-lactamase positive isolates had the *bla*Z gene confirmed by showing bands at 377 bp.

### Location of the *blaZ* gene in penicillin-resistant strains

Based on the Southern blotting, the *blaZ* gene was located on the chromosome in 27 isolates and on plasmids in 51 isolates. In 26 of the 34 beta-lactamase positive Finnish isolates (76%) and in 25 of the 44 beta-lactamase positive Swedish isolates (56.8%), the *blaZ* gene was located on a plasmid. The difference of the *blaZ* gene location between the Finnish and Swedish isolates was not statistically significant (p = 0.094).

Of the 27 isolates with chromosomally located *blaZ*, 12 (44.4%) were from clinical mastitis and 15 (55.6%) from subclinical mastitis. Of the 51 isolates with plasmid-located *blaZ*, 19 (37.3%) were from clinical and 32 (62.7%) from subclinical mastitis. The location of *blaZ* did not significantly differ between isolates from different type of mastitis (clinical vs. subclinical) (p = 0.629).

### PFGE analysis

The 147 *S. aureus* isolates showed 81 different pulsotypes. Twenty-seven, 18 and 16 isolates belonged to three dominating pulsotypes; the other pulsotypes included mainly one or few isolates. The most common pulsotype (28 isolates, 18 Finnish and 10 Swedish) included only beta-lactamase negative isolates. The pulsotype with 18 isolates (3 Finnish, 15 Swedish) included 16 isolates carrying the *blaZ* gene on a plasmid and two beta-lactamase negative isolates. The pulsotype with 16 isolates (7 Finnish and 9 Swedish) included 15 beta-lactamase negative isolates and one isolate carrying the *blaZ* gene on the chromosome. Most of the pulsotypes included few either beta-lactamase positive or negative isolates.

### Sequencing of the *blaZ* gene

The *blaZ* gene of 71 isolates (47 plasmid and 24 chromosomally located) of the total of 78 *blaZ* possessing isolates was successfully sequenced. Forty-seven plasmid-located and 6 chromosomally located *blaZ* regions were successfully amplified and sequenced using the primer pair 486–531. The length of the obtained sequences were 797 bp. Amplification of the chromosomally located *blaZ* regions of 18 isolates failed with these primers but succeeded using the primer pair 1F-1R, and sequences of 833 bp were obtained. In the further analysis, the region from 1 to 797 bp of the total *blaZ* gene of 846 bp of all 72 isolates was used. When the sequences were translated to proteins, amino acid sequences of 265 amino acids of the total length of 281 amino acids of the beta-lactamase protein were obtained.

Eighteen different amino acid sequences were obtained. Six different protein signatures, the signatures 1, 2, 3, 5, 6, and a new signature 2/5, which had similarities with the signatures 2 and 5, were found (Table 1). The most common signature type was the signature 3, including 24 plasmid and one chromosome located *blaZ*. The signature 6 included only chromosomally located *blaZ* (n = 17) and the signature 2 mainly plasmid located *blaZ* (15 plasmid, 1 chromosome). The protein signature of 7 isolates, including five chromosomally and two plasmid located *blaZ*, fell between the protein signatures 2 and 5, having >3 amino acid deviations from both. The protein signature of this group was similar (0 to 1 deviations) to the protein signature of *Enterococcus faecalis* with GenBank accession number U43087 [23], reported to belong to the protein signature 5 [18,19], but different (>3 deviations) from the other protein signature 5 references with accession numbers M25257, AY373761, AJ302698, and DQ919081.

**Table 1:** **Beta**-**lactamase protein signatures of the *blaZ *genes located chromosomally or on a plasmid in *S****. ****aureus *isolates in bovine mastitis in Finland and Sweden**

**Protein signature**	**Finland**		**Sweden**			
	**Chromosome**	**Plasmid**	**Chromosome**	**Plasmid**	**Total**	**Pulsotypes**
1				4	4	2 different pulsotypes
2	1	13		2	16	8 different pulsotypes
3		10	1	14	25	Two clusters of >64% similarity, with 8 and 4 different pulsotypes
5		1		1	2	2 pulsotypes with 78% similarity
6	4		13		17	9 different pulsotypes
2/5	3	1	2	1	7	A cluster of 4 pulsotypes with 81% similarity and 1 pulsotype with 61% similarity within the cluster
Total	8	25	16	22	71	

Fingerprints of the pulsed field gel electrophoresis (PFGE) with more than three band shifts were interpreted as being of a different pulsotype and thus the strains genetically unrelated.

The four isolates with the signature type 1 had an almost identical pulsotype, as had also the seven isolates with the signature type 2/5. The 25 isolates with the signature type 3 were divided into two groups of similar pulsotypes. The 16 isolates with the signature type 2 had rather similar pulsotypes but the 17 isolates with the signature type 6 had 11 different pulsotypes (Table 1).

We compared the beta-lactamase protein sequences of our isolates with the sequences submitted to the GenBank having a known beta-lactamase serotype or protein signature. The type B beta-lactamase corresponded to the protein signature 6, only found from isolates carrying the *blaZ* gene on the chromosome. The beta-lactamase type A corresponded to the protein signature 1, and the beta-lactamase type D to the protein signature 5, but representatives of beta-lactamase type C corresponded both to the protein signatures 2 and 3.

## Discussion

In most (76%) of the Finnish *S. aureus* isolates and in over half (57%) of the Swedish isolates *blaZ* gene was residing on a plasmid. The high proportion of penicillin-resistant *S. aureus* in Finland [10,12] could have been explained by plasmid-mediated resistance. However, nearly half of the Swedish isolates also had the gene on a plasmid but prevalence of penicillin resistant *S. aureus* has been low in Sweden [5,6]. Possession of the *blaZ* gene was partly linked to pulsotype, which may indicate a clonal spread of resistance. Under the selection pressure of antibiotic use, resistant populations of bacteria have advantage over susceptible ones and can become dominant. If possession of a resistance gene results in a fitness cost, resistant clones would disappear after cessation of antibiotic use. However, regarding *bla*Z-positive *S. aureus*, no evidence of a possible fitness cost is available as shown for some Gram-negative bacteria with other beta-lactamase genes [24]. It seems that other factors than location of the *blaZ* gene explain different prevalence of penicillin resistance among mastitis causing *S. aureus* in Sweden and Finland.

In spite of introduction of large scale mastitis control programs, *S. aureus* has remained a major mastitis pathogen in many countries [4,10]. *S. aureus* infection can persist for long periods in the mammary gland and needs to be treated with antimicrobials [25]. In human medicine, benzylpenicillin has lost its value as a therapeutic agent for treatment of *S. aureus* infections, because almost 100% of the isolates have been resistant for decades [26]. In isolates of human origin, the *blaZ* gene has been reported to be located predominantly on a plasmid [26]. For treatment of bovine staphylococcal mastitis, benzylpenicillin can still be regarded as a first-line antibiotic [2,27].

Many countries regularly monitor resistance of animal pathogens, including *S. aureus* isolates in bovine mastitis, by national monitoring programs. Based on the reports of these programs and other investigations, penicillin resistance of *S. aureus* is the most common form of antimicrobial resistance among mastitis causing bacteria [3,8]. In Finland, a nation-wide survey carried out in 2001 showed that the proportion of penicillin-resistant *S. aureus* isolates was 52% in subclinical mastitis [10]. In a more recent study on isolates from clinical mastitis, the proportion of resistance was only 25% [12]. The decreasing trend in penicillin-resistance of *S. aureus* could be due to mastitis control measures such as culling of cows infected with resistant isolates, but also due to the more prudent use of antibiotics [28]. However, the data are not fully comparable as the first originate from subclinical and the more recent from clinical mastitis. The differences in prevalence of penicillin-resistant *S. aureus* isolated in mastitis observed between countries may be explained by different management options to control *S. aureus* mastitis but possibly also by different use patterns of antimicrobials for mastitis treatment [15]. In Norway and Sweden, use of antimicrobials has been very restricted and benzylpenicillin has practically been the only antimicrobial used to treat mastitis over decades [27,29]. This indirect evidence may indicate that use of benzylpenicillin does not efficiently select for penicillin-resistance among mastitis causing *S. aureus*. Yet, systemic administration of penicillin or intramammary administration of dry cow penicillin-novobiocin combination were associated with penicillin resistance in Canadian dairy herds [7]. Similar results were reported also by Pol and Ruegg [30]. Together with restricted antimicrobial policy, strict culling of cows with mastitis caused by penicillin-resistant *S. aureus* has been advised for long in Sweden and in Norway. In Finland a wider selection of intramammary products containing broad-spectrum antimicrobials and combinations of different substances were earlier used [31]. In most countries, consumption figures of antimicrobials for different animal species are not available or not detailed enough, and no conclusions can be made on the associations between antimicrobial use and resistance.

The beta-lactamases of *S. aureus* have been classified immunologically by the serotype into four classes, types A-D [26]. The gene variant encoding the type B beta-lactamase is located on the chromosome whereas the gene variants encoding the types A, C, and D beta-lactamases are usually located on a plasmid [26]. These four beta-lactamase types correspond with the *blaZ* protein sequence signatures established by Olsen et al. [18], who reported 11 protein signature types for the *blaZ* sequences from staphylococci of different origin. Malik et al. [19] used the same protein signature classification and detected four known protein signature types and one new type from isolates from dogs and cats. We found five of these protein signatures, the signatures 1, 2, 3, 5 and 6, and a new signature, which had similarities both with the signatures 2 and 5 and was named 2/5. The majority of our isolates with the *blaZ* gene in the chromosome were of the protein signature type 6, which clustered with the beta-lactamase protein sequences from the GenBank having the beta-lactamase serotype B. The protein signature 6 was detected only in isolates with chromosomal location of the *blaZ* gene. This is in concordance with earlier studies [26]. The new protein signature type 2/5 was found in seven isolates (4 Finnish and 3 Swedish), of which five carried the *blaZ* gene on the chromosome. The pulsotypes of these isolates were very similar, indicating clonal spread. The protein signature 1 corresponded with the beta-lactamase type A, and the protein signature 5 with the beta-lactamase type D. The protein signatures 2 and 3 corresponded with the beta-lactamase type C. With two exceptions, the protein signatures 1, 2, 3 and 5 were found in plasmid located *blaZ,*which is in agreement with other studies [18,26].

Certain genotypes of mastitis causing *S. aureus* can become dominant in the dairy herds [25]. In the three most common pulsotypes here *bla*Z-negative isolates were over-represented, indicating that penicillin-resistance was partly related to pulsotype. An association between certain pulsotypes and penicillin susceptibility has also been shown previously [32,33]. Penicillin-resistance may be linked to other virulence factors of *S. aureus*, which may facilitate the spread of resistant clones [33]. Intramammary infection remained significantly more often chronic if it was caused by *bla*Z positive (61.0% remained persistent) than *blaZ* negative (25.0%) strains [34]. In Denmark, penicillin resistance in mastitis causing *S. aureus* in 1950s was exclusively found in isolates of the same phagetype and ribotype, but found among several clones in 1992 [35]. The authors suggested that penicillin-resistance was introduced to bovine *Staphylococcus* population first by one clone but then became widespread among several clones, probably due to horizontal transfer. According to a Norwegian study, 99 of 107 penicillin and tetracycline resistant *S. aureus* isolates in 18 herds belonged to the same clone [36]. Location of the resistance genes was not determined but the resistance was suggested to have spread through a single clone and a resistance plasmid.

## Conclusions

Presence of the *blaZ* gene of *S. aureus* was pulsotype-linked. Six different protein signatures were found and all except one both in Finnish and Swedish isolates. The protein signatures were not clearly associated with certain pulsotypes. The *blaZ* gene was located on plasmids in more than half of the isolates. The plasmid location of the *blaZ* gene was not statistically significantly more common in Finland than in Sweden, and hence does not explain the higher penicillin-resistance in *S. aureus* causing bovine mastitis in Finland compared to Sweden.

## Competing interests

The authors declare that they have no competing interests.

## Authors’ contributions

AFB performed the laboratory analyses and drafted the manuscript. ST helped in laboratory and statistical analyses and drafted the manuscript and performed the analysis of sequences. SP designed and coordinated the study and drafted the manuscript. BB and A-LM participated in study design and helped to draft the manuscript. All authors read and approved the final manuscript.
